# Acceptability of adding a non-contrast abdominal CT scan to screen for kidney cancer and other abdominal pathology within a community-based CT screening programme for lung cancer: A qualitative study

**DOI:** 10.1371/journal.pone.0300313

**Published:** 2024-07-01

**Authors:** Juliet A. Usher-Smith, Golnessa Masson, Angela Godoy, Sarah W. Burge, Jessica Kitt, Fiona Farquhar, Jon Cartledge, Michael Kimuli, Simon Burbidge, Philip A. J. Crosbie, Claire Eckert, Neil Hancock, Gareth R. Iball, Suzanne Rogerson, Sabrina H. Rossi, Andrew Smith, Irene Simmonds, Tom Wallace, Matthew Ward, Matthew E. J. Callister, Grant D. Stewart

**Affiliations:** 1 Department of Public Health and Primary Care, University of Cambridge, Cambridge, United Kingdom; 2 Department of Oncology, University of Cambridge, Cambridge, United Kingdom; 3 Department of Surgery, University of Cambridge, Cambridge, United Kingdom; 4 Leeds Teaching Hospitals NHS Trust, Leeds, United Kingdom; 5 Faculty of Biology, Medicine and Health, University of Manchester, Manchester, United Kingdom; 6 Leeds Institute of Health Science, University of Leeds, Leeds, United Kingdom; 7 Faculty of Health Studies, University of Bradford, Bradford, United Kingdom; Makere University College of Health Sciences, UGANDA

## Abstract

**Objectives:**

The Yorkshire Kidney Screening Trial (YKST) is a feasibility study of adding non-contrast abdominal CT scanning to screen for kidney cancer and other abdominal malignancies to community-based CT screening for lung cancer within the Yorkshire Lung Screening Trial (YLST). This study explored the acceptability of the combined screening approach to participants and healthcare professionals (HCPs) involved in the trial.

**Methods:**

We conducted semi-structured interviews with eight HCPs and 25 participants returning for the second round of scanning within YLST, 20 who had taken up the offer of the additional abdominal CT scan and five who had declined. Transcripts were analysed using thematic analysis, guided by the Theoretical Framework of Acceptability.

**Results:**

Overall, combining the offer of a non-contrast abdominal CT scan alongside the low-dose thoracic CT was considered acceptable to participants, including those who had declined the abdominal scan. The offer of the additional scan made sense and fitted well within the process, and participants could see benefits in terms of efficiency, cost and convenience both for themselves as individuals and also more widely for the NHS. Almost all participants made an instant decision at the point of initial invitation based more on trust and emotions than the information provided. Despite this, there was a clear desire for more time to decide whether to accept the scan or not. HCPs also raised concerns about the burden on the study team and wider healthcare system arising from additional workload both within the screening process and downstream following findings on the abdominal CT scan.

**Conclusions:**

Adding a non-contrast abdominal CT scan to community-based CT screening for lung cancer is acceptable to both participants and healthcare professionals. Giving potential participants prior notice and having clear pathways for downstream management of findings will be important if it is to be offered more widely.

## Introduction

Kidney cancer is largely curable if detected early. However, up to 60% of patients are asymptomatic at the time of diagnosis [1] and over 25% are diagnosed with metastatic disease [2] which carriers a poor prognosis. Together with the increasing incidence of the disease [2], this has led to increasing calls from both the scientific community and patient representatives for further research around the potential for introducing kidney cancer screening programmes [3–7]. The hope is that early detection of asymptomatic kidney cancer would stage-shift the disease to a lower stage at diagnosis, reduce the prevalence of later-stage disease and in turn reduce kidney-cancer mortality. Current evidence suggests that kidney cancer meets many, but not all, of the criteria for screening. However, the benefit of earlier initiation of treatment remains unknown and uncertainty also exists about the optimal screening modality and target population [7]. Given the relatively low prevalence of kidney cancer, targeted screening of high-risk individuals and/or combining kidney cancer screening with screening for other conditions, is likely to be the most cost-effective strategy.

Following a number of successful trials, lung cancer screening programmes with low dose chest CT (LDCT) have started being introduced in many high-income countries. This has in turn led to interest in the potential for combining abdominal CT scans to screen for kidney cancer and other upper abdominal pathology with the LDCT scans within lung cancer screening programmes [8]. Combining the abdominal CT scans with LDCT within lung cancer screening programmes has the major advantage of reducing the need for separate appointments and so reduces the burden on participants and the cost to the healthcare system. The rates of kidney cancer are also higher in the subset of the UK population eligible for lung cancer screening [9], in part due to the shared increased risk associated with cigarette smoking which is one of the best-established risk factors for development of kidney cancer [10].

The Yorkshire Kidney Screening Trial (YKST) [11] is a feasibility study nested within the Yorkshire Lung Screening Trial (YLST) [12] in which participants returning for their two-year follow-up appointment are offered an additional non-contrast abdominal CT scan to screen for kidney cancer and other abdominal pathology alongside a LDCT. As well as assessing the feasibility, logistics and clinical outcomes of offering of an abdominal CT scan alongside screening for lung cancer, a key objective of YKST is to assess the acceptability of the combined approach to both potential participants and healthcare professionals. As well as informing the design of future studies in this area, the importance of assessing acceptability is reflected in the published criteria used to assess screening programmes, which state explicitly that there needs to be evidence that each programme is clinically, socially and ethically acceptable to health professionals and the public [13, 14].

While the uptake of screening programmes provides a quantitative measure of one component of acceptability, acceptability is a multi-faceted construct that reflects the extent to which people delivering or receiving a healthcare intervention consider it to be appropriate, based on anticipated or experienced cognitive and emotional responses to the interventions [15]. The aim of this embedded study was, therefore, to use qualitative methods to assess in-depth the acceptability of adding the non-contrast abdominal CT scan from the perspective of the potential participants, both those who accepted the abdominal scan and those who declined it, and healthcare professionals involved in both YKST and YLST.

## Materials and methods

### Ethical approval

This study was granted approval by the North West—Preston Research Ethics Committee (reference 21/NW/0021)

### Design

A semi-structured qualitative interview study.

### Participant recruitment

Participants were recruited from the Yorkshire Kidney Screening Trial (YKST) (NCT05005195 and ISRCTN18055040). Full details of the trial are described elsewhere [11]. In brief, YKST is a feasibility study of adding a non-contrast abdominal CT scan to screen for kidney cancer and other abdominal pathology to a LDCT within YLST, a trial of community-based CT screening for lung cancer [12]. Within YLST, individuals at high risk of lung cancer are offered lung health checks, including LDCT screening for lung cancer, at mobile units in community locations at baseline (T0) and again two years later (T2). Between May 2021 and October 2022 participants attending the mobile units for their T2 visit who had not had an abdominal CT within the previous six months and had not had a previous diagnosis of kidney cancer were invited to take part in YKST.

Potential participants were informed of YKST on arrival at the mobile unit and invited to listen to a YKST information video immediately after the YLST T2 Patient Information video. The decision to inform participants at this point and not prior to arrival at the mobile unit was taken to minimise any impact of the additional abdominal scan on uptake to YLST. The YKST video explained the context of the study and the benefits and harms of the additional abdominal CT. Specifically, potential participants were informed that there were no direct benefits from having the scan itself but that in about 5 out of 1000 people being offered the abdominal scan, the scan may show evidence of kidney cancer and that the scan may also pick up problems in other organs, including the liver, pancreas and aorta. The potential for overdiagnosis was included by saying that “cancers picked up through screening tend to be early and more treatable but it has not yet been proven in trials that detection of these cancers by screening reduces deaths”. Potential risks from the additional abdominal CT scan were additional radiation (the extra dose being estimated as the same amount of background radiation over a 12-month period), the potential for tests or treatments for findings that were not needed and the potential to cause anxiety and worry. Participants were also provided with a written participant information sheet covering the same information (S1 File). Translation services were offered to patients where required. Participants were then asked if they would like to take part in YKST and consent taken.

Those who accepted and those who declined the additional abdominal scan were also asked if they consented to be contacted by a researcher to discuss taking part in a qualitative research interview about reasons for agreeing or declining participation. Those consenting to be contacted were sent a separate participant information sheet and consent form and asked to contact the team if they would like to take part. A member of the research team then purposively sampled those who accepted the scan based on age and sex and contacted them, and all those declining the scan, to arrange a convenient time for a telephone or remote video interview. Participants for this study were recruited between 10 May 2021 and 19 July 2021.

All healthcare professionals involved in the screening process were also invited to take part in an interview about the acceptability of the combined approach and the logistics. With the exception of the radiographers who were asked only about their specific role in performing the abdominal CT scans, all were members of the wider YKST research study team and so were familiar with all aspects of the study. All were provided with a separate participant information sheet and consent form for this interview study. Participants were invited to take part between August and November 2022 and interviews took place between 19 August 2022 and 25 January 2023.

### Consent

Those participants taking up the abdominal scan within YKST provided written consent to take part. Separate consent was taken from all participants who took part in this sub-study. That consent was either written or verbal, with verbal consent obtained at the start of the interviews by asking: ‘Could you please state for the record that you are happy for this interview to be audio-recorded?’. The interviewee’s response was audio recorded and included in the interview transcription.

### Screening process

Participants consenting to the additional abdominal scan completed consent and a short baseline questionnaire within a YLST consultation and were then taken to the CT scan room on the mobile unit where they had the YLST thoracic LDCT, followed immediately by the YKST abdominal CT. Scans were reported by a team of uro-radiology consultants within 14 days and categorised as normal (YKST1) or abnormal (YKST2-5). Participants with a normal scan were sent a letter from the research team explaining that their scan was normal and that there were no further actions required. All abnormal scans were discussed in twice-weekly screening review meetings and participants sent letters explaining the findings and any further actions recommended. Participants with potentially serious findings were also telephoned by the research nurse.

### Data collection

Semi-structured Interviews were conducted remotely using Teams or by telephone by one of two researchers (GM, an academic GP with qualitative experience and AG, a trial manager with many years’ experience of data collection). The interview schedules were informed by the Theoretical Framework of Acceptability (TFA) [15]. The TFA consists of seven component constructs: affective attitude, burden, intervention coherence, ethicality, opportunity costs, perceived effectiveness, and self-efficacy. Interviews with study participants explored their views on the acceptability of the combined screening approach, the consent process, the information provided about the additional scan, their reasons for accepting or declining the scan and their thoughts around the potential benefits and harms of the additional scan. Interviews with healthcare professionals focused on the aspect(s) of the study that each healthcare professional had been involved in, including where relevant views on acceptability of the study processes, including obtaining consent, ensuring only participants consenting to the additional abdominal scan receive it, performing, reporting and reviewing the additional scans and feeding back the results. All interviews were recorded and transcribed for analysis.

### Analysis

The data were analysed using thematic analysis, guided by the TFA, and supported by NVivo v12 software. For the analysis of the data from participants, a coding frame was initially developed and refined by two authors (GM and JUS) based on reading and re-reading the transcripts. Both researchers coded four transcripts against the final coding frame to ensure agreement and provide triangulation of perspectives on the data. The remainder of the transcripts were then coded by one researcher (JUS). The codes were then brought together into a broad set of themes which were mapped onto the seven component constructs of the TFA (affective attitude, burden, intervention coherence, ethicality, opportunity costs, perceived effectiveness, and self-efficacy). The themes under each construct were then discussed amongst a multidisciplinary group of the wider research team and the final themes and sub-themes agreed by consensus. The analysis of the data from the HCPs took place after the analysis of the participant data. After familiarisation with the data, HCP interview transcripts were coded directly into the constructs of the TFA by one researcher (JUS). Agreement was then checked by a second researcher (AG). For both the YKST participants and HCP analysis, care was taken throughout the analysis to consider how the perspective and involvement in the research process of each member of the research team might affect the findings. This included ensuring that the individuals who had conducted the interviews were involved throughout all stages of the analysis and their notes on the interviews incorporated. As many of the interviews with the HCPs had been conducted by a trial manager who had worked closely with the HCPs throughout the trial, the HCP primary analysis was also led by a researcher experienced in qualitative research who had been less involved with the day-to-day running of the trial and so brought a different perspective to the data analysis.

## Results

We conducted interviews with a purposive sample of 20 YKST participants who had taken up the additional abdominal scan, five YLST participants who had declined the additional scan and eight healthcare professionals involved in the screening process (three research nurses, two consultant urologists, a consultant radiologist, a radiographer and a research administrator). Details of the YKST and YLST participants are given in Table 1.

**Table 1:** Characteristics of YKST and YLST study participants.

Characteristic	YKST participants accepting the scan	YLST participants declining the scan
	*n = 20*	*n* = 5
**Age (years)**		
55–59	3	0
60–64	5	1
65–69	2	2
70–74	5	0
75–79	5	2
**Sex**		
Male	10	3
Female	10	2
**Ethnicity**		
White British	18	4
Asian	2	0
Other	0	1
**Index of multiple deprivation quintile**		
1 (most deprived)	3	1
2	3	1
3	5	2
4	6	1
5 (least deprived)	3	0
**Aware of results of scan at time of interview**		
Yes	6	---
No	14	---
**Scan result (if known at time of interview)**		
Normal	4	---
Abnormal	2	---

The findings from the interviews with all three groups are presented below under each of the seven component constructs of the TFA. Participants are identified by whether they accepted (A) or declined (D) the scan, their self-reported gender and, for those who had accepted the scan, whether they had not received their results prior to the interview (no results) or received a normal or abnormal scan result (normal/abnormal). To reduce identifiability of the HCPs who took part in the study we have attributed quotes throughout the text to an HCP participant number rather than specific roles.

### Ethicality

Participants who took up the offer of the additional abdominal scan described feeling ‘*privileged*’ (A01, female, no results) and ‘*lucky*’ (A02, female, no results) to have been offered it, with the decision to have the scan described as a ‘*no brainer*’ by several (A04, female, no results). The decision to accept the scan had been an instant decision for almost all and had been made at the point at which they were invited, before hearing any of the potential benefits or harms of the scan:

As soon as it said you offered a kidney scan, are you willing? I just said Yes I wasn’t really bothered about the blurb because I was already convinced I was going to say ‘Yes’. (A10, male, no results)

then when I was offered the kidney scan I jumped… Yeah. I said ‘Do everything else while you are at it’ that’s my philosophy. You know while I’m in there if you can look at everything I’ve got get on with it; I just think it’s marvellous that I had this opportunity. (A11, female, no results)

*Often people say to me it is a bit a no brainer*, *you are getting checked*, *it makes sense to get it done*, *to get it checked*. (HCP4)

For the majority of these participants (n = 11), the main reason behind their decision to take up the scan was a belief that it is always better to know and if there was the potential to find something earlier then that was always a good thing and would directly benefit them (Table 2). The lack of proven benefit from finding conditions earlier was not raised as a consideration. Others (n = 6) were motivated by potential benefits to the wider NHS due to decreased costs treating conditions earlier, by a desire to help others through research or by just feeling that taking part was ‘*the right thing to do*’. Three participants were directly motivated by concerns about their kidneys. The altruistic nature of many of these decisions was also reflected in the interviews with HCPs, with one observing that ‘*the overall majority just say ‘Well I might as well while I am here and if it doesn’t help me it will help somebody else’ there’s certainly an element of altruism in the patients that are coming*.’ (HCP1)

**Table 2:** Reasons for taking up the scan and illustrative quotations.

Reason	Illustrative quotations
Belief that it is better to know	*Well anything that can pick up an earlier cancer has got to be a good thing hasn’t it and I knew you had found some incidentals so*.* *.* *. *so why not*. *(A15*, *female*, *normal)* *Like I said if there is anything they can find out early enough or nothing at all being better but I would like to know as soon as I could rather than getting ill and then finding out it was my kidneys without a clue*. *(A19*, *male*, *no results)* *I’m up for anything that is going to detect anything at an early stage*. *(A18*, *female*, *no results)*
Helping others through research	*I like the thought of possibly helping somebody else in the future*, *it could even be my own children or my grandchildren*. *I just think it is great to be able to do these trials*. *(A11*, *female*, *no results)* *…and the fact that maybe they would do a national screening depending on the results on these things that you are doing at the moment for other people as well*, *not just for me*. *(A02*, *female*, *no results)*
Sense of duty	*So it just seemed to me it was the right thing to do…*..*I think this is something that people get offered to do should do it not just for themselves but for your family as well*, *future generations*. *(A01*, *female*, *no results)* *I have faith in the NHS and what it does*. *If somebody turns around and says to you ‘Just try this or this may help you’ etc*., *then you are going to take it*. *(A07*, *male*, *no results)*
Better for the NHS	*And it makes services available for other people cheaper in the sense that if something is picked up early and is treated it doesn’t go on to develop and take up additional resources*. *…*.*I think it is a win win*, *you see it is better for the National Health Service and its certainly better for the patients*. *(A10*, *male*, *no results)* *I mean I would rather sooner catch something at a very beginning maybe a cancer than actually waiting for symptoms and then going down the route of maybe more invasive*, *and also cost the NHS more money in that respect as well*. *(A17*, *female*, *no results)*
Concern about kidneys	..*this made me think about my kidneys having had the kidney stones a few years before*. *So when you mentioned the kidney scan and I thought oh yes I will have that*. *(A16*, *male*, *no results)* *I am aware that I have got dodgy kidneys so it was good thing to do*. *(A14*, *male*, *abnormal)* *Basically because I have been poorly for such a long time and I don’t know what is wrong and er*.* *.* *. *Because I cough quite a bit I do get a lot of pain in my kidneys etc*., *so no it was good to have it there and to be offered it*. *(A07*, *male*, *no results)*

Of the five participants taking part in this study who had declined the scan, one had not understood that he had been offered it. Similar to those accepting the scan, the other four participants who had knowingly declined the scan, had all made the decision on being initially informed of the opportunity and before hearing or reading the participant information:

*[I made the decision not to have the scan] right at the word ‘Go’ really*. […] *Well you see this is the thing isn’t it because*… *It was suddenly thrust upon me for want of a better word and you know I like to process things in my head and think about the process I am about to go through*, *and I had only done that for the lungs based on what my experience was the first time*. (D01, female)

The reasons for declining the scan included worry about what it might find from a participant with only one kidney, insufficient time to make a decision and uncertainty over forthcoming investigations Table 3.

**Table 3:** Reasons for declining the scan and illustrative quotations.

Reason	Illustrative quotations
Worry about what it might find	*You know like you think leave well alone; just leave things well alone and leave things as they are*.*[…] I just said ‘No*. *I don’t want to know*. *I am not interested’ […] If it showed anything else then I feel I would be panicking like nowt on earth*. *(D03*, *female)*
Insufficient time to make a decision	*it just hit me so quick I didn’t think myself about that*, *you know I’ll just leave it for now while I get other things sorted*. *(D04*, *male)* *Because I was unprepared I kind of backed off; you know when in doubt refrain*. *If I had known about it before [the visit] my response would have been different*. *(D01*, *female)*
Uncertainty over forthcoming investigations	*Well my initial thought was it was a good thing to do and under normal circumstances I would have consented and done it*. *However I had a problem because at the time I’d erm*.* *.* *. *I had quite serious infection a couple of weeks previously and I was on a fast track to see the Urology department at the hospital who and as far I am aware they were going to do screening for me there*. *(D02*, *male)*

### Affective attitude

All those who had taken up the scan spoke very positively about their experiences, with all feeling that they had made the right decision to take up the abdominal scan. The main area discussed was whether there had been sufficient time to make a decision about whether to take up the additional scan or not. Linked with how almost all had made their decision immediately on hearing about the option, all but one of those accepting the scan reported that they felt they had had sufficient time to make a decision.

Well certainly [there was enough time to think about it] for myself because it was an instant decision in the first place. (A20, male, no results)

Several did comment, though, that others might have appreciated more time and, in particular, might have appreciated knowing in advance:

I mean now that you are mentioning it I wonder if when I got the letter for the lung test I wonder if they should have mentioned it then or included some literature. That doesn’t bother me but maybe some people would have liked to have known beforehand before they turned up that they were going to be offered that maybe….Yes I think now that we are talking about it I think so, I think it would have been better. (A04, female, no results)

Similarly, the process on the mobile unit was described as efficient, with those accepting the scan saying there was ‘*ample opportunity*’ (A17, female, no results) for questions and they were ‘*not rushed at all*’ (A19, male, no results) but one participant described how it was an ‘*industrial sort of process*’ and might have prevented some individuals from feeling able to ask questions.

I didn’t feel the need to ask questions but I am conscious that it was a hard industrial sort of process… There were no real gaps in the process. I sat down for about two minutes other than that I was moved through. Now that suits me I was quite happy with that but I can see where some people might be a bit wary about being sort of hurried through to the scan room, and […] may well feel I don’t know a bit uneasy about starting to ask questions at that stage. (A16, male, no results)

This was reflected in the experiences of one of those who declined the scan who regretted having not taken up the offer of the additional scan even before leaving the mobile unit but did not feel comfortable raising her concerns:

Well with hindsight… When I went in to have the scan I probably should have asked the radiologist. But at that point I had made up my mind I thought well I can’t now suddenly reverse this, or maybe they would have been agreeable to that. But I mean it is all happening isn’t it? You know there’s other people queuing behind you and that kind of thing, so you just sort of think I just want to be in and out of here as quick as possible. (D01, female)

Notably three of the other participants who had declined the scan also regretted their decision.

All the HCPs spoke very positively about their experiences of the study. In particular, adding the abdominal scan within an existing community-based lung screening trial was viewed as an advantage, especially for recruitment, and was considered to fit well into the existing processes:

*We were very very lucky*, *the Lung Screening Trial was well established*, *a really big complicated trial that we could just tag onto that*. *And it has been remarkable with what feels like hardly any effort at all we’ve been one of the biggest recruiting trials in the UK for anything this year*. (HCP5)

*You know it fitted; you were able to fit [the consent] into the consultation quite easily*. *What I tended to go through the YLST stuff first to make sure was okay there because they need to be in YLST to be in YKST and I kind of slotted that in towards the end of the YLST stuff*. (HCP4)

The risk of jeopardising the success of the lung screening trial was recognised though, with some HCPs feeling under additional pressure, particularly at the start of the study:

*One of the difficult things at the beginning was knowing that YLST was already an established Trial and was very successful*. *So we didn’t want to do anything to jeopardise the success of that by anything that we might have done to upset the patients*, *or made them feel uncertain about coming back again for more lung follow ups*. (HCP3)

### Burden / opportunity costs

Having the additional abdominal scan was considered very little burden to participants:

Well I was more than happy just to do it to be honest; it wasn’t going to hurt which was the main thing. It wasn’t going to take up a great more time anyway so yeah not a problem. (A08, female, normal)

And because it was told that it was just like an add on to the scan I was getting anyway I wouldn’t notice any difference, I wouldn’t have to get up and go onto another scanner, it wouldn’t stop it will just carry on, so an extra few minutes. (A19, male, no results)

Consistent with the finding that most had made the decision to take up or decline the scan before hearing or reading the information sheet, the potential risks associated with the scan had little impact on their decision.

Of the three main risks (radiation exposure, need for unnecessary investigations, and the potential for anxiety and worry), the additional radiation was recalled most frequently. The majority (n = 16) of participants who took up the scan and both of those who had declined but watched the information recalled mention of radiation as a risk to having the scan. Only one of those who took up the scan was concerned about the additional radiation though. The others either dismissed or minimised the risk by comparison with other risks or prior exposure, considered the additional exposure insignificant or, notably, trusted that the researchers and the NHS would not be offering the scan if it were a significant risk to them (Table 4).

**Table 4:** Views on radiation dose and illustrative quotations.

Reason	Illustrative quotations
There are lots of other risks	*I seem to remember something about minimal or some such word*. *But like I say things like that don’t bother me*. *You know let’s be honest it’s a risk breathing in the air with all the pollutants so something that is going hopefully*, *benefit your future; I just think any risk is better than the risk of cancer*. *(A11*, *female*, *no results)* *You don’t really measure it in your mind there are lot of things we are dosed with unknown in our life which in fact is doing us more harm than radiation*. *There are things on food that we eat and the things that we handle in the garden and in the workplace that we will regret years later*. *(A03*, *male*, *normal)*
Prior exposure to radiation	*I’ve had a kidney scan in the past; I’ve had ones where they inject things MRIs and various other x-rays for broken bones and various other things*. *I don’t think about oh blimey I am going to get some extra rad here if I am not careful*, *I will get a total that is going to make me ill I just keep going*. *I am 75 I’ve survived quite a few scans I don’t really get worried about them*. *(A16*, *male*, *no results)* *No [the radiation did not concern me] because I wanted it to be done*. *I’ve had radiotherapy before for cancer so er*.* *.* *. *I’ve already had huge doses throughout my life*. *(A14*, *male*, *abnormal)*
Trust in the study	*To be honest I didn’t give it a great deal of thought I just thought well you are hardly going to be doing this if it is going to be incredibly dangerous for people*. *(A08*, *female*, *normal)* *I would have thought that if it was a problem they wouldn’t do the scan if it was such a great risk*, *I put my trust in them*. *(A17*, *female*, *no results)* *I have my faith in the NHS I don’t think they would put me at risk…*.. *I didn’t really think too much about it*. *(A04*, *female*, *no results)* *The fact is if it was so dangerous you wouldn’t be doing these things*. *(A11*, *female*, *no results)*
Seemed a small insignificant risk	*Well it is no more than the background radiation that you normally pick up*, *you can go some parts of the country Cornwall for instance and you get a bigger dose than from an x-ray*. *(A10*, *male*, *no results)* *Vaguely he said about radiation is harmful but the amount you are going to be given is neglible*, *it wouldn’t make any difference but they have to tell you anyway*. *(A19*, *male*, *no results)* *There was something about the radiation risk and it seemed like any risk you know*.* *.* *. *to the average normal healthy person was exceptionally low*. *(D02*, *male)*
Benefits outweigh the risk	*Yes it equivalent to 12 month outside does seem high*. *I did think that at the time but it still didn’t put me off*. *You know I think the benefits outweigh the risks hopefully*. *(A15*, *female*, *normal)* *you know that it is radiation but er*.* *.* *. *I think the positives outweigh the negatives by so much I think*, *for me anyway*. *(A06*, *male*, *no results)*

Only a minority of participants (n = 4) recalled the need for unnecessary tests or investigations being mentioned. To those participants, having additional tests that may turn out not to have been necessary was considered ‘*part and parcel*’ (A09, female, no results) of having the scan and ‘*something you deal with if it happens*’ (A06, male, no results). After being reminded of the potential risk of unnecessary tests or investigations in the interviews, there was an overwhelming view that knowing was always better, including amongst those participants who had first-hand experience of potentially unnecessary treatment:

Well like I say I’d rather know than not know. The fact is if they have found something, anything, nothing, whatever… Not that I want any more surgery but at the end of the day I’d rather have surgery than suffer long term consequences of maybe something. (A11, female, no results)

Only three participants who had accepted the scan recalled anxiety and worry as potential harms. None had been concerned by that as a risk at the time. When the potential risk for anxiety and worry was raised in the interviews, the majority did not feel that was a concern for them and described how they had ‘*not given a thought to it*’ since the scan (A01, female, no results and A18, female, no results). Several participants did, however, acknowledge some anxiety whilst waiting for the results or the potential for anxiety when receiving the results.

It will be a relief when I get my letter to say No everything is fine. So there’s probably a tiny tiny little thing thinking oh I hope nothing comes up but not a great worry to me. (A02, female, no results)

The burden of adding the abdominal scan on the lung screening trial and wider healthcare system was discussed by all HCPs. The three areas with the greatest impact were the process of ordering the scans which was described as ‘*onerous*’ and required two separate scans to be requested for each participant, the need to use and learn three IT systems, and the additional clinical workload generated downstream. The concerns around additional clinical workload had generated mixed feelings amongst some of the clinical teams, with unnecessary referrals for findings that were already known about and the reporting of insignificant abnormalities particularly cited:

*We’ve made repeated referrals that are unnecessary for people that are already on the screening programme*, *and maybe causing those patients anxiety and causing extra workload for the vascular team*. *There is an issue about national v local record keeping and finding*… *you know we make a finding that is already known somewhere but not to us*. (HCP5)

*I think the biggest thing for me is that we’ve recorded all abnormalities found be they significant or not and that’s created a lot more work downstream*. *So recording an abnormal renal mass or an adrenal enlargement or erm*… *pancreatitis*, *that’s fair and that’s valid*, *and they all need a valid clinical review*. *But the radiology reporting reported renal cysts for example which are really common and each of those needed an initial review and then need a six month review and that’s created additional work*. (HCP5)

*I think its mixed feelings*… *I think everyone viewed it as positive there is a lot of*… *you know there’s always some kind of YKST generated stuff to do that I wouldn’t have been doing otherwise but then*… *But several people who had big tumours that wouldn’t have been found otherwise*, *who may well have become untreatable had they been left at the same time*. (HCP7)

Features of the study which had helped minimise these impacts were good communication, both between the lung screening and kidney teams and with other speciality teams, and the use of template letters for communication of results.

### Intervention coherence

The rationale for combining the abdominal scan with the lung scan made sense to all but one participant, including all those who had declined the scan. They could see benefits in terms of efficiency, cost and convenience both for them as individuals and also more widely for the NHS which provides free healthcare to all residents of the UK (Table 5). These benefits of combining the scans were also echoed by the HCPs, although most HCPs qualified their statements with whether there is proven benefit to patients.

**Table 5:** Views on combining abdominal and lung scanning and illustrative quotations.

Views on combined screening	Illustrative quotations
More efficient	*I mean I just feel if you are having one area scanned you know it must make it much more efficient to scan other areas surrounding at the same time*. *(A17*, *female*, *no results)* *Its brilliant I mean for very little extra effort and money you are getting a double whammy*. *(A02*, *female*, *no results)* *I think it is an excellent idea considering it uses the same machine why not do it all at once*. *(D02*, *male)* *It’s certainly more efficient*. *I mean if you are already doing a CT scan it doesn’t take much more time for the patient or the staff to do an additional scan*. *So I think if there is a proven benefit yes it is a very sensible way of doing it*. *(HCP1)*
Saves money	*Well I think it’s a good idea because if the whole process has been set up and paid for to do the lung screening and it*.* *.* *. *I am not saying it won’t cost much more but there must be an economic benefit there of doing more than one screening together*, *especially for research purposes*. *(A05*, *female*, *normal)* *Oh yes it has bound to be financially better isn’t it for the NHS and more convenient for the patient*. *(A04*, *female*, *no results)*
Saves time	*It is just by combing everything not only are you saving the NHS or the Trial people’s time*. *You know it is one person who sees you*, *you get your interview*, *you read your leaflets or you look at your video*, *you get organised have your scan and they always ask you is there any questions and whatever else and then you go*. *(A11*, *female*, *no results)* *It also saves people waiting for another CT scan or scan*, *it saves time basically*. *(D05*, *male)* *I mean you have got them once so if you can do two for one*, *if you like the time and effort involved to do both compared to one is probably minimal*. *So it is a good idea if it makes sense from a wider point of view for a number of patients you need to screen to find any cancers*. *(HCP7)*
Convenient	*I think it makes a lot of sense you are lying there you are going up and down in the tube and then they said ‘Now we are going to do your kidneys’ and you just go up and down a couple of times which is fine*, *it very convenient*. *Better than having a separate appointment to go*. *(A14*, *male*, *abnormal)* *It is just the convenience and you haven’t got to wait for something it is all done in one day and if I had one of them and then the next week I had another one you would be thinking*.* *.* *. *but it is all done in one day it is just better*. *(A19*, *male*, *no results)* *I think it is a very good idea it’s convenient for everybody involved all round*. *You are already there*, *the machine is already there it is already in use for the sake of a few more minutes do a bit more with it*. *(A20*, *male*, *no results)* *And I am sure for the costs to the NHS*, *it must surely be convenient to them as well so*.* *.* *. *to do it in one hit rather than setting up one for this and one for that*. *(D01*, *female)* *The results may come back and say otherwise because I don’t know what the final outcome would be but the radiation dose and the time taken is [*?*9*.*46 so low]*. *I think it is worthwhile*. *(HCP6)*

The one participant who did not see the rationale for the combination was one who had accepted the scan but described initially being quite shocked to be offered the additional scan and, even after the interview, didn’t understand why it was being offered:

Well I didn’t know why I was being offered the scan at first erm… you know and even now it’s the relationship between the lungs and the kidneys I don’t… But to be offered the whole thing at once, yeah I was quite shocked. Quite shocked. (A07, male, no results)

Almost all participants, including the two who had declined the scan but watched the video, felt they had had sufficient information about the scan and understood the purpose of the study

[I] completely [understood] because she laid it all out everything like the positive and negative and it was very clear, you could not make a mistake at what she was saying, so you couldn’t misunderstand anything really. (A06, male, no results)

The video was very comprehensive and also they gave me written notes as well so you can never say [anything negative] about it. It was straightforward, easy to understand, not alarming or anything. (A10, male, no results)

Despite this there was evidence both from the participants and from the HCPs of two areas of misunderstanding. The first was that the scan would look at organs other than the kidneys. While this was obvious to some:

It was obvious it was going to be more than just the kidneys because the kidneys are surrounded cheek by jowl by everything so you were going to get the full picture, you weren’t going to narrow it down just to my kidneys it was going to be a big area. (A09, female, no results)

For others the fact that the scan would cover other organs had not been clear in the information. One felt it ‘*seemed to hint at [a check on other organs] in the video*’ (A16, male, no results) but had not appreciated that other organs would deliberately be looked at and three others had not realised at all. Which other organs were nearby or would be visualised was also unclear.

*But I think one of the things now that we know again about our findings*, *we can make patients more aware that we don’t always just find cancer*, *we can find other things within the abdominal area*. (HCP3)

The second area of misunderstanding was around what a CT scan of the kidneys might find and how that differs from having blood tests to monitor kidney function. In particular, several participants described how they had regular blood tests to monitor their kidney function with their GP and were not clear what a scan would add. This was perhaps related to a general lack of awareness of kidney cancer and greater familiarity with kidney failure:

To be honest I’ve never come across anybody having kidney cancer. I’ve heard of them kidney failure and dialysis and all that and kidney failure and but cancer… whether it has got something to do with cancer that they have dialysis or what… (A13, male, abnormal)

*Discussions varied really about the radiation they might be exposed to*, *about having monitoring by the GP for the kidneys*. *But often that wasn’t necessarily the structural kidney*, *it was the functioning so they would collect like the U’s and E’s and things like that*, *so people misunderstood*. (HCP4)

### Perceived effectiveness

The absolute number of people who may benefit from the scan did not appear important to either those who had taken up the scan or those who had declined it. Only one participant could recall the risk estimate given in the participant information video. Half recalled that there had been an estimate of the number of people who may have findings but could not recall the number and described how it had not influenced their decision about whether to take up the scan or not:

No it [the number] didn’t influence me at all I would have made the same decision to go ahead with the scan….. even if it just picked up one it would be beneficial (A20, male, no results)

The others did not recall a number being given at all and after being given the number in the interview, even those who thought the number was low dismissed the importance of knowing the absolute benefit:

No I don’t recall it but then again I tend to fade out when people starting quoting figures (A16, male, no results)

If they had said one in a million I would still have had it done. (A10, male, no results)

### Self-efficacy

Although self-efficacy was not directly relevant to the participants in this study as they had already attended for the CT scan when making the decision of whether to take part or not, 18 of the 20 participants would have taken up the kidney scan if offered at a different time from the lung scan. There was also a view that combining the abdominal scan within lung screening might potentially encourage others to take part:

I think it’s a good idea and they should do more of it, because it is difficult enough to get hold of people to be screened or scanned. (A03, male, normal)

I think it’s a good idea […] possibly encourage people to have both scans, to have the two scans whereas if you say well ‘I will do the lung scan now and we will do a cancer scan at some other time’, and then they might sort of think oh I can’t be bothered doing that one I will just do this one. (A08, female, normal)

## Discussion

### Key findings

Overall, combining the offer of a non-contrast abdominal CT scan alongside the low-dose CT within a community-based lung cancer screening programme was considered acceptable to all the participants of this study, including those who had declined the abdominal scan. In particular, the rationale for combining the abdominal scan with the lung scan made sense and fitted well within the process, and participants could see benefits in terms of efficiency, cost and convenience both for themselves as individuals and more widely for the NHS. The scan itself was also considered low burden by participants, with almost all participants making an instant decision about whether to accept the scan or not at the point of invitation. These decisions were based more on trust and emotions than the information provided, with the potential benefits or risks having little impact. Despite this, there was a clear desire for more time for people to make a decision. Concerns were also raised about the burden on the study team and wider healthcare system arising from the additional workload both within the screening process and downstream following findings on the abdominal CT scan.

The support seen here for the combined approach is consistent with a survey of 1,562 adults in Australia in which 85.3% (CI 81.9–88.2%) stated they would support a combined ‘One Stop’ cancer screening programme [16]. Our findings also show that almost all participants, including those who declined the abdominal scan, made an instant decision based on their prior beliefs and were not influenced by the absolute number of people who may benefit or the potential harms. This is consistent with the wider literature around screening and decision-making that shows how decisions around screening are often influenced by emotions and attitudes and not by quantitative risk-based information [17–23]. As in those studies, the main reason most participants gave for taking up the abdominal CT scan, was related to a belief that it is always better to know and that if there was the potential to find something earlier then that was always a good thing and would directly benefit them. As long as there was the potential for one person to benefit, the absolute number was not important to participants.

Similarly, there was little concern about the potential harms. In particular, despite it being mentioned specifically in the participant information leaflet, only a minority of participants recalled the potential for additional unnecessary tests, and even after being reminded of that as a possible consequence in the interviews, none considered it a concern. Instead, even those who had had first-hand experience of the negative consequences of additional tests and investigations, considered it ‘part and parcel’ of screening and outweighed by the potential benefits. This lack of concern about harms has been reported previously [24] and highlights the challenges with ensuring participants provide informed consent for screening. A new finding from this study is the role that trust played in the decisions participants made and in their views of the combined approach. Many of the participants in this study described how they trusted the research team and the wider NHS not to offer anything to them that would be harmful, and by extension that any further investigations done following any findings on the scan would be in their best interests. Understanding and considering the potential harms (or benefits) themselves was, therefore, not necessary as that responsibility lay with others. Similarly, some participants were motivated to take up the scan by a wider social perception that participant was ‘the right thing to do’. This sense of moral obligation to participate in screening has been seen in the wider screening and social science literature [25–32] and, again, highlights both the widespread positive perceptions of screening amongst the public and the challenges with informed consent in this context.

### Strengths and limitations

To our knowledge this is the first study to report on the acceptability of combining abdominal and lung CT scans within the context of screening. A key strength is our use of the theoretical framework of acceptability as a framework through which to explore acceptability. This approach meant we considered acceptability not as a single construct but as one which is multi-faceted. As a result, we were able to explore what individuals both thought about the combined screening approach as a whole as well as what they felt about the different components. The value of this analysis approach is particularly evident amongst those who declined the additional abdominal scan. In these individuals we were able to describe their reasons for not taking part and also their positive attitudes towards combining abdominal CT scanning within lung cancer screening programmes. Had we not considered the multiple constructs of acceptability, it might have been easy to conclude that combining abdominal CT scanning with lung cancer screening was not acceptable to these individuals.

A further strength is the inclusion of both participants who accepted and declined the abdominal CT scan and healthcare professionals. This enabled us to explore acceptability from a range of perspectives and, crucially, to identify some of the impacts within the wider healthcare system. The participants in this study were, however, already contributing to or participating within a screening trial for lung cancer and attending the two-year follow-up visit and so are a group of individuals already engaged with research and screening. Our findings may not, therefore, reflect the attitudes of individuals outside this context and the acceptability both for participants and for HCPs may vary with changes in how the screening and results pathway is designed. The comments in relation to the benefits of the combined approach to the NHS may also not be applicable internationally in different healthcare settings, particularly as the NHS makes people proud to be British and is considered crucial to British society [33] but during the period of this study the system was widely known to be struggling in terms of capacity and funding following the COVID-19 pandemic. The lack of ethnic diversity further reduces the transferability of our findings. We also did not assess acceptability of those not eligible for the additional abdominal scan. Previous work suggests that restricting access to screening based on smoking status may be less acceptable than eligibility determined by non-modifiable factors [34] and so this group will be an important group to include in future studies.

### Implications for research and policy

The findings from this study have a number of implications both specifically for combining abdominal CT scans within lung cancer screening and also for cancer screening programmes in general. Firstly, the rationale for combining the abdominal scan with the lung scan made sense to participants and was viewed almost universally positively. However, the instant decision-making and reliance on trust within the wider healthcare system highlight the importance for policy makers to only offer appropriate and evidence-based screening. It is not sufficient to simply include details of potential harms and expect members of the public to use that information to make an informed decision over whether to take part or not. This includes the consideration of over-diagnosis which, at least with the materials and descriptions used in this study, is not well understood. Secondly, while incorporating the additional scan was generally considered to fit well, the potential extra workload both for those healthcare professionals directly involved and downstream in the wider healthcare system are important considerations. The three areas that generated the greatest burden on the system were the process of ordering the scans, the need for staff involved in the delivery of the study to use and learn three IT systems, and the additional clinical workload generated as a result of clinical findings on the abdominal scans. If abdominal CT scans are to be introduced alongside lung screening programmes the IT processes will need to be addressed and careful consideration given to the handling of insignificant findings. This will involve the need for clear clinical pathways and potentially new streamlined pathways for the most common findings. Quantitative data on the prevalence of findings within the YKST study will help to inform these. Thirdly, despite the instant decision making for most, there was clear articulation of a need for additional time for people to decide whether to take up the scan or not, particularly amongst those who declined the scan and later regretted that decision. Alternative pathways for informing participants of the option in advance will need to be developed.

## Conclusion

Together, our findings suggest that combined screening programmes, specifically in this case combining abdominal CT scanning within lung screening programmes, is likely to be widely acceptable to both participants and those healthcare professionals involved in the programmes, particularly if there are clear streamlined pathways for the most common clinical findings and mechanisms for informing participants in advance of the offer of the additional scan. However, while acceptability is necessary for the success of any screening programme, demonstrating acceptability is not sufficient to suggest that abdominal CT scans should be offered within such programmes. Further data are needed to quantify the potential benefits of early detection, the impact of incidental findings, and the overall cost-effectiveness of the approach. The clinical findings and health economic analysis from YKST will provide some of these data. This study shows that if the next step of a large randomised controlled trial is considered appropriate, such a trial would likely be acceptable to the public and those involved in delivering the screening and subsequent clinical care.

## Supporting information

S1 File Participant information sheet. (PDF)
