# Supplementary material for: Acceptability of adding a non-contrast abdominal CT scan to screen for kidney cancer and other abdominal pathology within a community-based CT screening programme for lung cancer: A qualitative study
Source: PLoS One. 2024 Jul 1;19(7):e0300313. doi: 10.1371/journal.pone.0300313 (PMC11216619; doi:10.1371/journal.pone.0300313)
Supplement: S1 File — (PDF) [file pone.0300313.s001.pdf]

Yorkshire Kidney Screening Trial
Patient Information Sheet

Thank you for your interest in taking part in the Yorkshire Kidney Screening Trial today and having an additional CT scan of your kidneys at the same time as your lung scan. This leaflet gives you more information about the study. A video with the same information as this leaflet is available if you would prefer this.

What will happen if I decide to take part?
If you would like to, you can have an additional CT scan of your kidneys at the same time as your lung scan today. Whilst going through the lung health check questionnaire with you, a member of the research team will ask if you are interested in also being part of the kidney screening study. If you are, the researcher will go through the information in this leaflet and check you understand what will happen and are happy to take part. We will then ask you to sign a consent form and answer some additional questions about your kidney health. When you have your lung CT scan, you will also have the extra scan of your kidneys. This will happen immediately following the lung CT and take an extra 10-15 seconds. You will not need to change position. The scan is still pain-free and you will not need an injection. Trained staff will be present and they will talk you through what is happening. The whole lung and kidney health checks including the scans will still take less than one hour.

You will also be asked whether you would be happy to potentially be contacted by a member of the research team at a later date to take part in a phone or video interview. The purpose of the interview will be to talk about how you felt being offered an additional kidney CT scan and why you did or did not choose to have the extra scan. If enough people have already taken part in these interviews by the time you come for your lung health check, you may not be contacted by the research team even if you say you would be happy to take part in an interview.

Why are you offering me a Kidney CT scan?
The kidney CT scan is looking for any changes in your kidneys that might need treatment. One of the particular things we are looking for are any early signs of kidney cancer. Checking for cancer in this way is called screening. You may have heard of screening for other types of cancer such as breast cancer or bowel cancer.

Kidney cancer is the 8th most common cancer in Yorkshire. It has a poor survival rate, with only 6 out of 10 patients diagnosed with kidney cancer still alive after 5 years. This is partly because many people with kidney cancer don't have any symptoms. Kidney cancer is often not diagnosed until the disease has passed the point at which we can easily cure it. Screening for kidney cancer has the potential to pick up these cancers earlier and increase the number of people who can be cured.

There are a variety of tests which could be used to screen for kidney cancer. These include a CT scan of the kidneys. It would not be appropriate to perform a CT scan just to look for signs of kidney cancer. However, in this study because you are already having a CT scan of your lungs, we are offering you the opportunity to add on a CT scan to look for kidney cancer.

For all types of cancer screening, there are benefits but also some downsides. We would like to explain these to you so that you can make up your own mind and decide for yourself if you would like the extra scan.

What are the potential benefits of the extra CT scan?

There is no direct benefit from having the scan itself. However, we believe that there could be some benefit if early, more treatable, cancers are detected. We estimate that in about 5 out of a 1000 people the scan will show evidence of a kidney cancer. These cancers picked up through screening tend to be early and more treatable. However, it has not yet been proven in trials that detecting these cancers by screening reduces deaths – this study is the first step in gathering the evidence to see if this is the case.

The CT scan may also pick-up findings in the kidney which are not cancer but which require further tests or a repeat scan in a few months' time.

Although the extra CT scan is being done to look at the kidneys, it will also include your upper abdomen. This means we may pick up problems in other organs, including the liver, pancreas and aorta. If such problems are detected, you will be referred to the appropriate specialist to diagnose and treat them.

Are there any risks from the extra CT scan?

CT scanners use radiation to produce the pictures of your kidneys. Exposure to radiation can itself cause problems (very rarely actually causing cancer), but by using very modern CT scanners we can reduce the amount of radiation needed. The extra CT scan of the kidneys will slightly increase the amount of radiation you are exposed to. This extra dose is the same amount of radiation that we come into contact within our daily lives over a 12 month period. The likelihood of this scan detecting an early cancer is far greater than the likelihood of the scan causing you harm.

Occasionally, people may have tests or treatments for findings that were not needed. This is because the finding later turns out to be benign (not a cancer) or is a harmless type of kidney cancer (that would not cause problems even if left alone). It is important you are aware of this possibility before having the scan.

Having the extra CT scan of your kidneys could make you feel anxious or worried about what the scan might show. If a problem is found needing further tests or treatment, this may also cause you worry and anxiety. If you would like to talk to our doctors and nurses they will always be available to discuss any concerns you may have.

What will happen after the scan?
An expert team of doctors will check your CT scan. We will write to you with your results within the next 4 weeks. If you need any other tests or treatment you may need to come to a hospital clinic. Sometimes these tests include extra scans or biopsies. The team of doctors will make sure you are only sent for tests that are necessary. We will take your wishes into account at every step of the process. All decisions about tests or treatment will be made jointly by you and your doctor or nurse.

How accurate is CT screening?
CT scanning is very accurate but not perfect. Very occasionally, the screen will give a normal result, but will fail to pick up an existing cancer. It is very important that you tell your GP if you develop any new symptoms after the CT scan, for example abdominal pain, blood in the urine or unintentional weight loss. If necessary your GP can arrange extra tests.

Is CT screening currently available on the NHS?
CT screening of the kidneys is not yet available on the NHS. We need more evidence before we can decide whether the NHS should offer kidney screening. The Yorkshire Kidney Screening Trial is helping to provide this evidence.

Why have I been invited?
We are inviting all participants in the Yorkshire Lung Screening Trial who attend for their scheduled Lung Health Check follow up to have the additional kidney scan.

Will my information be kept confidential?
Yes. Only the study team at Leeds Teaching Hospitals, the University of Leeds, the University of Cambridge and other healthcare professionals who need access to your medical records will know you are taking part in the study. We will ask if you are happy for us to access your medical records, as well as contact your GP and other doctors about your health both during and for 10 years following the study and to keep this information securely for 15 years. This will allow us to see how any findings identified by the kidney CT scan are managed, and what happens to participants involved in the study over a long period of time. If you agree, we will also keep your GP informed of the results of the additional kidney scan. If you agree to be contacted about potentially taking part in a phone or video interview, then your name and contact details may be passed onto researchers at the University of Cambridge. That information will be stored securely.

We plan to share study information with other researchers, including those at the University of Cambridge, but we will not share any data that identifies you. We will publish the results of the study in medical journals, but again you will not be identified. If you agree we will keep your CT scans and other linked information about you for use in future research projects. These projects might not involve the original research team and could be either an academic or commercial partner. We will make sure that anyone we share data with has permission for their research and we will use the minimum data necessary.

The University of Leeds is the sponsor for the study which means they are responsible for the research. The University and Leeds Teaching Hospitals' are joint data controller for the study. This means they will look after your data and make sure that it is used properly. This will include checking that the information collected about you is accurate. Only the people who carry out the checks will see information about you. The information stored in the University will include your NHS number and Date of Birth, but will not include your name, address or telephone number. No one from the University will contact you as part of this checking process.

You can stop being part of the study at any time, without giving a reason. We will then stop collecting new data about you but we will keep information about you that we already have.

You can find out more about how we use your information by contacting the research team using the contact details at the end of this form or the University of Leeds data protection officer at: ####

More information can also be found by visiting:
www.hra.nhs.uk/information-about-patients/
https://dataprotection.leeds.ac.uk/research-participant-privacy-notice/
http://www.leedsth.nhs.uk/patients-visitors/patient-and-visitor-information/how-weuse-your-data/

What will happen to the results of the research study?

- At the end of the study a summary of our results will be published on the study website which can be accessed at www.ykst.org.uk.
- The results of this research will inform recommendations about how the NHS delivers cancer screening tests.
- The results will be reported in scientific journals and presented at academic conferences. While we may use your quotes, you will never be identified.

Thank you for taking the time to read this information sheet and for thinking about taking part. Taking part in this trial is purely voluntary and the additional kidney scan will not happen unless you have signed the accompanying Patient Consent Form.
